# Alterations in Lymphocytic Metabolism—An Emerging Hallmark of MS Pathophysiology?

**DOI:** 10.3390/ijms24032094

**Published:** 2023-01-20

**Authors:** Viktoria B. Greeck, Sarah K. Williams, Jürgen Haas, Brigitte Wildemann, Richard Fairless

**Affiliations:** 1 Department of Neurology, University Clinic Heidelberg, 69120 Heidelberg, Germany; 2 Clinical Cooperation Unit (CCU) Neurooncology, German Cancer Consortium (DKTK), German Cancer Research Center (DKFZ), 69120 Heidelberg, Germany

**Keywords:** autoimmunity, glycolysis, lymphocytes, metabolism, mitochondria, multiple sclerosis, oxidative stress, peripheral blood mononuclear cells

## Abstract

Multiple sclerosis (MS) is a chronic autoimmune disease of the central nervous system (CNS) characterised by acute inflammation and subsequent neuro-axonal degeneration resulting in progressive neurological impairment. Aberrant immune system activation in the periphery and subsequent lymphocyte migration to the CNS contribute to the pathophysiology. Recent research has identified metabolic dysfunction as an additional feature of MS. It is already well known that energy deficiency in neurons caused by impaired mitochondrial oxidative phosphorylation results in ionic imbalances that trigger degenerative pathways contributing to white and grey matter atrophy. However, metabolic dysfunction in MS appears to be more widespread than the CNS. This review focuses on recent research assessing the metabolism and mitochondrial function in peripheral immune cells of MS patients and lymphocytes isolated from murine models of MS. Emerging evidence suggests that pharmacological modulation of lymphocytic metabolism may regulate their subtype differentiation and rebalance pro- and anti-inflammatory functions. As such, further understanding of MS immunometabolism may aid the identification of novel treatments to specifically target proinflammatory immune responses.

## 1. Introduction

Multiple sclerosis (MS) is a chronic autoimmune disease of the central nervous system (CNS) characterised by acute inflammatory demyelination and neuroaxonal degeneration in the white and grey matter of the brain and spinal cord [1]. The prevalence of MS is steadily increasing with around three million people affected worldwide in 2020, predominantly women [2,3]. First symptoms manifest in early adulthood, and the majority of patients (around 85%) are diagnosed with the relapsing–remitting form of the disease (RRMS), in which recurrent episodes of acute neuroinflammation that trigger neurological symptoms alternate with periods of complete or partial recovery [4]. Most RRMS patients eventually transition to a secondary progressive stage (SPMS) where disability increases steadily as a result of ongoing neurodegeneration [4]. A minority of patients are diagnosed with primary progressive MS (PPMS), which is characterised by steady disease progression without recovery from disease onset [4]. The rate of disease progression, and the type and severity of symptoms vary considerably between patients. Depending on the CNS lesion site, these symptoms include impaired motor, sensory and cognitive functions, as well as fatigue, pain and depression [5].

Although its aetiology remains incompletely understood, MS is thought to be triggered by an interplay of genetic susceptibility and environmental factors. Thus, a large number of gene variants, mostly linked to immune system function, as well as smoking, low vitamin D levels and Epstein–Barr virus infection have all repeatedly been associated with increased disease vulnerability [6,7,8]. These genetic and environmental risk factors are thought to contribute to aberrant immune system activation in the periphery and subsequent migration of autoreactive, proinflammatory lymphocytes to the CNS where they contribute to inflammatory and neurodegenerative processes [9]. This migration is facilitated by a disturbance in the blood–brain barrier allowing their extravasation and accumulation within the CNS. However, while repeated mass infiltration of T and B lymphocytes into the CNS across this compromised barrier is typical for RRMS, progressive forms of the disease are characterised by a slow accumulation of lymphocytes in the absence of detectable blood–brain barrier permeability. It has been proposed that the presence of these cells in progressive disease may have originated from earlier subclinical disease activity prior to the reestablishment of the barrier [1]. During the acute inflammation characteristic of RRMS, activated T and B cells contribute to axon demyelination both directly and indirectly through the release of proinflammatory cytokines, deposition of autoantibodies, and the recruitment/activation of additional immune cells, including microglia and macrophages. However, in progressive forms of the disease, both immune-dependent and independent pathological processes have been described, although with potential interplay. These include the formation of ectopic B cell follicles, the activation of innate immune cells, mitochondrial dysfunction, oxidative stress and glutamatergic excitotoxicity resulting in ionic imbalances in areas of chronic demyelination that ultimately drive axonal loss and neurodegeneration [1,10]. Furthermore, the variation in immune responses within the CNS between relapsing and progressive disease forms may reflect differences observed between their immune cell compositions and activation profiles in the periphery [11,12,13].

To date, a range of immunosuppressive and immunomodulatory drugs have been approved for the treatment of MS. These significantly reduce relapse rates in RRMS by counteracting acute inflammation, but do not slow neurodegeneration-induced disease progression in SPMS and PPMS and often have considerable side effects [14,15]. Gaining a better understanding of the underlying pathophysiological processes in MS is, therefore, crucial for the development of more effective treatments.

In addition to inflammation, both metabolic and mitochondrial dysfunctions have recently emerged as important features of MS pathophysiology and restoring normal mitochondrial functioning as well as addressing the balance between glycolysis and oxidative phosphorylation (OxPhos) could represent a viable novel treatment target [16]. To date, most research has focused on the role of mitochondrial impairment in CNS degenerative processes (see Section 2 below) but a small number of studies suggest that alterations in mitochondrial activity are also present in immune cells of MS patients and in murine models of the disease (see Section 4). Considering the central role of these cells in MS pathophysiology and the crucial importance of lymphocyte metabolic processes in their activation and function (see Section 3), this is an intriguing avenue of research. Following a brief summary of cellular energy production and the role of mitochondrial dysfunction in CNS pathology, this review provides a comprehensive overview of recent research assessing metabolic changes in MS lymphocytes and the targeting of these processes in animal models of MS.

### 1.1. Production of Cellular Energy

**Figure 1:**
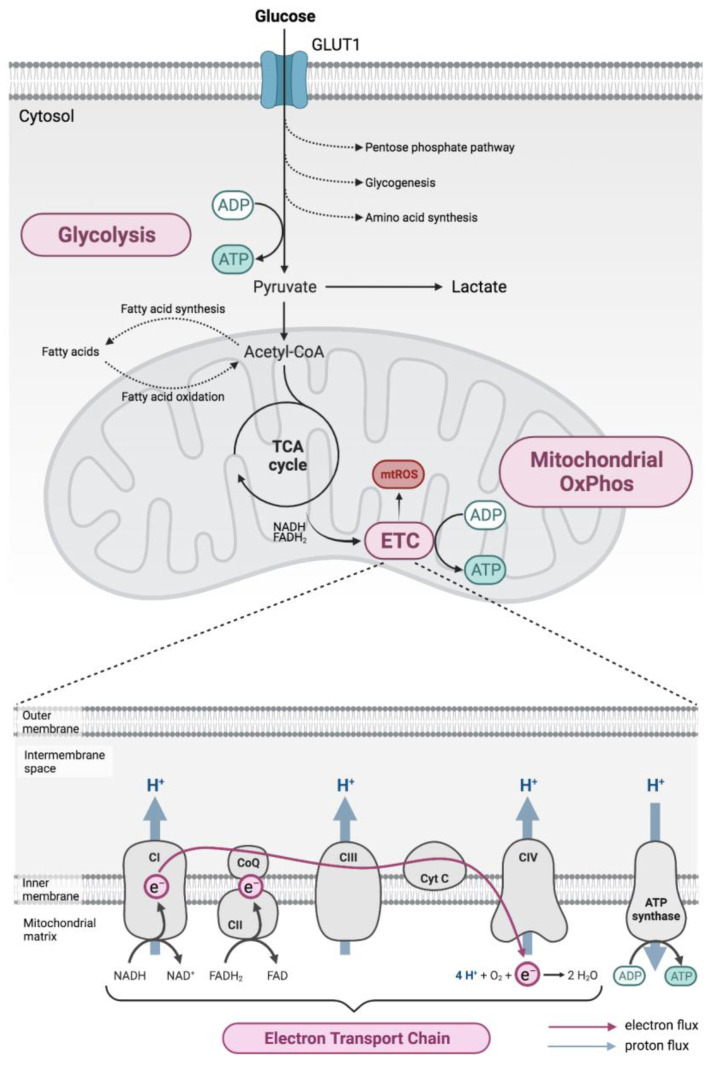
Cellular metabolism. Energy production principally involves glucose metabolism via glycolysis, which is closely coupled to the mitochondrial respiratory chain, which is located at the inner mitochondrial membrane and consists of electron transport chain (ETC) complexes I (CI), II (CII), III (CIII) and IV (CIV), as well as ATP synthase (complex V, CV). Energy released during electron (e^−^) transport along the ETC (pink arrow) allows protons (H^+^) to be moved to the intermembrane space (blue arrows), generating an electrochemical gradient that is used by complex V to synthesise ATP. ADP: adenosine diphosphate; ATP: adenosine triphosphate; e^−^: electron; FAD^+^: flavin adenine dinucleotide; FADH_2_: dihydroflavin adenine dinucleotide; H^+^: proton; H_2_O: water; NAD^+^: nicotinamide adenine dinucleotide; NADH: dihydronicotinamide adenine dinucleotide; O_2_: oxygen; OxPhos, oxidative phosphorylation; Pi: inorganic phosphate; TCA, tricarboxylic acid.

Energy metabolism involves a complex interplay of metabolic pathways, which are highly regulated in order to meet the varying cellular energy requirements arising from rapid changes in the cell’s environment or functions. Neurons have a particularly high energy demand since they require ATP to drive many homeostatic processes such as restoring ionic gradients following axonal action potentials or calcium influx at synapses [17]. Similarly, activated T and B lymphocytes require high amounts of energy to drive the production of the many macromolecules needed during active proliferation as well as the production of cytokines. The metabolic pathways employed by different cells are, however, very similar and typically involve glycolysis, the tricarboxylic acid (TCA) cycle, the pentose phosphate pathway and finally, OxPhos in the mitochondria (see Figure 1).

Briefly, glycolysis involves the metabolic conversion of glucose through several intermediates to pyruvate resulting in a net production of two ATP molecules for each glucose molecule, as well as reducing two NAD+ to NADH. Pyruvate then undergoes oxidation before feeding into the TCA cycle, generating in total four NADH, one FADH2 and one GTP per pyruvate molecule. The TCA cycle occurs in the mitochondrial matrix, with pyruvate being carried across the inner mitochondrial membrane from the cytosol under aerobic conditions. Reduced NADH and FADH2 are then used as substrates to drive OxPhos in the mitochondria generating more of the main energy substrate, ATP [18].

Under anaerobic conditions, as well as aerobic conditions when a high energy burden exists, rather than directing pyruvate towards the TCA cycle, it is instead metabolised to lactate by the enzyme lactate dehydrogenase. During this conversion, NADH is consumed generating NAD+, which can be reused to drive further glycolysis allowing more glucose to be metabolised and ATP to be generated. This allows energy to be produced quickly through glycolysis and is used to supply energy during short-term but intense periods of activity, such as rapid cell proliferation. Lactate is subsequently secreted, and eventually converted back to pyruvate in the liver by a process known as the Cori cycle [19].

### 1.2. Role of Mitochondria in Energy Production

Mitochondria are complex organelles found in all eukaryotic cells. They consist of an outer and highly folded inner membrane and contain their own DNA (mtDNA) that encodes several RNAs and proteins. Although they are involved in a number of essential cellular processes, including calcium homeostasis and apoptosis, one of their most important functions is the production of energy in the form of ATP via OxPhos [16].

Under normal physiological conditions, electrons donated by the reducing agents NADH or FADH2 (generated during glycolysis and the TCA cycle) are transferred to oxygen via a series of oxidation–reduction reactions taking place in the electron transport chain (ETC). This is located at the inner mitochondrial membrane and consists of a set of multisubunit complexes [20] acting as electron carriers (Figure 1). Complex IV, the last enzyme in the ETC, then reduces oxygen to water, thereby consuming around 90% of cellular oxygen. The energy that is released during electron transport along the ETC is used by complexes I, III and IV to move protons across the inner mitochondrial membrane to the intermembrane space against their concentration gradient [21]. Together, these processes give rise to the proton motive force, an electrochemical gradient across the inner mitochondrial membrane that consists of an unequal distribution of protons and an electric potential (the mitochondrial membrane potential) [22]. This proton motive force subsequently drives protons back into the matrix through complex V, and the energy that is released is used to synthesise ATP from ADP and inorganic phosphate (Pi) [22]. In this way, OxPhos couples electron transfer along the ETC and ATP production at complex V thus forming the mitochondrial respiratory chain. In addition to ATP, the respiratory chain produces reactive oxygen species (ROS) as by-products of normal mitochondrial metabolism [23]. While ROS serve important signalling functions under normal physiological conditions, excessive ROS production due to mitochondrial dysfunction or impairments in endogenous antioxidant defences may result in oxidative damage to the mitochondria or to the cell as a whole [23,24].

A number of experimental approaches can be used to evaluate mitochondrial function, including the assessment of the oxygen consumption rate (OCR), ETC complex activity, mitochondrial membrane potential or mitochondrial ROS production (for a review see [21,22]). Changes in the glycolytic activity, on the other hand, can be indirectly assessed by, for example, measuring the extracellular acidification rate (ECAR), which reflects, at least in part, the glycolytic production of lactate, the expression of rate-limiting glycolytic enzymes such as hexokinase or the expression of glucose transporters such as GLUT1 [25]. Used in combination, these approaches have provided a detailed understanding of mitochondrial and glycolytic activity in neurons and in peripheral lymphocytes of MS patients and murine MS models.

## 2. Mitochondrial Dysfunction Is Involved in CNS Degenerative Processes in Multiple Sclerosis

**Figure 2:**
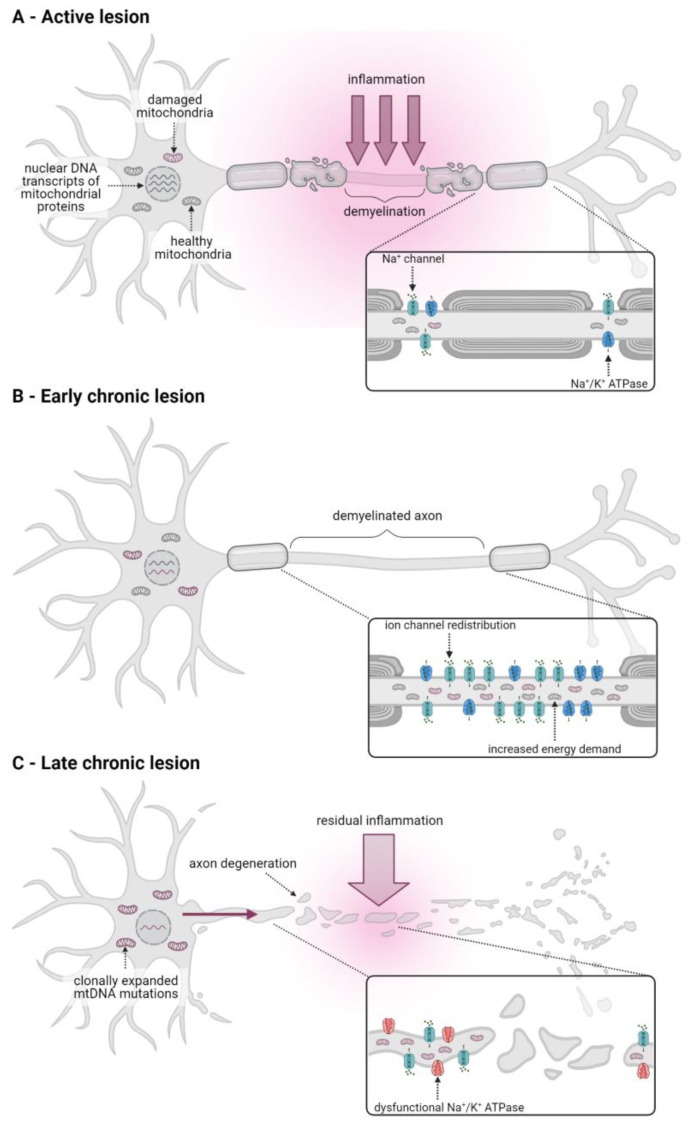
Mitochondrial dysfunction contributes to CNS pathology in all stages of MS. (**A**) In active white matter lesions of acute MS, inflammatory mediators released by activated immune cells induce mitochondrial dysfunction and contributes to axonal demyelination. (**B**) The redistribution of sodium channels in demyelinated axons in chronic inactive lesions of progressive MS patients results in an increased energy demand due to an upregulation of sodium/potassium (Na^+^/K^+^) ATPase activity. A subsequent increase in mitochondrial number and activity protects the demyelinated axon against energy deficiency-induced degeneration. (**C**) Over time, mitochondrial damage in the axon accumulates, both due to increased ROS production by damaged mitochondria and residual inflammation in the vicinity of the axon and due to the supply of dysfunctional mitochondria from the soma (pink arrow) that is caused by reductions in nuclear DNA transcripts of mitochondrial proteins and clonal expansion of mtDNA mutations. This results in axonal energy deficiency, Na^+^/K^+^ ATPase failure, and subsequent intra-axonal ionic imbalances that trigger degenerative pathways.

Neurons, and especially axons, require large amounts of ATP to maintain key functions, such as action potential transduction, which is dependent on the activity of sodium/potassium (Na^+^/K^+^) ATPases that restore ionic gradients and thus the resting membrane potential [26]. This dependency on OxPhos renders neurons particularly vulnerable to mitochondrial dysfunction. Accumulating evidence indicates that mitochondrial alterations, including mtDNA mutations and ETC dysfunction, are an important feature of CNS pathophysiology across all stages of MS [27,28] and contribute to white and grey matter degeneration (recently reviewed in [29]; Figure 2).

In active white matter lesions, which are a hallmark of RRMS and acute progressive MS and are characterised by severe inflammation and ongoing axonal demyelination, the expression of mtDNA, as well as nuclear DNA-encoded OxPhos genes, particularly ETC complexes I and IV, are markedly downregulated [30]. Decreased complex I and IV expression and activity has also been observed in axons in chronically active lesions and in pattern III lesions, a subtype of acute white matter lesions associated with hypoxia [31,32,33]. Inflammatory mediators, such as proinflammatory cytokines (for example, tumour necrosis factor α) and ROS released by immune cells, are thought to contribute to this dysfunctional mitochondrial OxPhos activity, which in turn exacerbates inflammation-induced tissue damage by inducing a state of neuronal energy deficiency [26,30,31] (Figure 2A).

Acute inflammation eventually subsides but a proportion of axons fail to remyelinate, thus forming chronic inactive lesions [16]. In progressive MS patients, a large proportion of these demyelinated but otherwise intact axons are characterised by an increase in axonal mitochondrial mass and an upregulation of mitochondrial proteins and complex IV activity [33,34,35]. This results from the redistribution of sodium channels along the demyelinated axon segments and upregulation of Na^+^/K^+^ ATPase activity in order to maintain physiological intra-axonal ion concentrations along this greater axonal surface area in the absence of myelin. In turn, this homeostatic upregulation of Na^+^/K^+^ ATPase activity results in an increased energy demand in the demyelinated axon [27,36]. It has, therefore, been suggested that the observed increase in axonal mitochondrial content and OxPhos activity in demyelinated axons may be compensatory mechanisms that ensure efficient activity of the Na^+^/K^+^ ATPase required to maintain ionic homeostasis, thereby protecting the axon against degeneration [37] (Figure 2B).

In the long term, however, the increase in OxPhos activity and production of damaging ROS by dysfunctional mitochondria in demyelinated axons, together with any residual inflammation, cause damage to the ETC resulting in inadequate ATP supply [37]. The subsequent Na^+^/K^+^ ATPase failure, in turn, leads to intra-axonal ionic imbalances that trigger degenerative pathways that result in slowly advancing axonal degeneration in chronically inactive lesions. Contrary to active lesions, degeneration in these lesions is largely independent of inflammation, but contributes to neurological decline in progressive MS patients [27].

In addition to mitochondrial alterations in white matter lesions, mitochondrial dysfunction has also been observed in the grey matter of progressive MS patients. Thus, neurons in the motor and frontal cortices and in the cingulate gyrus showed a decreased expression of nuclear DNA-encoded ETC transcripts and markedly reduced activity of complexes I, III and IV [38,39,40,41]. The reduced cortical expression of nuclear-encoded OxPhos genes may be due to alterations in their transcriptional control as it was accompanied by a decrease in the transcriptional co-factor peroxisome proliferator-activated receptor-gamma coactivator (PGC)-1α, an important regulator of OxPhos gene-related transcription factors, and a reduced expression of the transcription factor complex containing nuclear respiratory factor (NRF)-2 [16,41,42]. Additionally, neurons deficient in complex IV activity were found to carry high levels of clonally expanded mtDNA mutations [39]. These mutations may result from oxidative and inflammation-induced damage and contribute to mitochondrial dysfunction once their expression levels exceed the expression of wild-type transcripts [16,39]. In addition to changes in gene expression, mitochondrial respiratory chain complexes in the grey matter may also be directly impaired by inflammatory mediators, such as cytokines and ROS secreted by activated immune cells [26].

Overall, the resulting accumulation of dysfunctional mitochondria in the grey matter of progressive MS patients has been shown to correlate with neuronal loss [41]. In addition, this exacerbates the energy deficiency of demyelinated axons in the white matter since the grey matter soma can no longer supply these with healthy mitochondria that they would need to meet the increased energy demands associated with demyelination [37] (Figure 2C).

In sum, there is now considerable evidence suggesting that mitochondrial dysfunction in the white and grey matter contributes to neurodegeneration and clinical disease progression in all stages of MS but particularly in progressive patients. However, recent research has also uncovered alterations in mitochondrial metabolism in immune cells, which play an important role in MS pathophysiology.

## 3. Lymphocyte Activation and Their Role in MS and EAE

### 3.1. Lymphocytic Metabolism under Non-Autoimmune Conditions

As part of their natural function, lymphocytes are activated in response to infections or cancers resulting in a range of responses including proliferation and the production of pro- or anti-inflammatory cytokines, amongst others, which in turn allow the direct killing of pathogens, as well as infected or mutant cells. Maintaining these responses requires a continuous supply of energy and, as has been well-studied in the case of T cells, their activation is, therefore, critically dependent upon the reprogramming of their cellular energy metabolism (for a recent review see for example [43]). Resting T cells differentiate upon stimulation into distinct T cell subpopulations including, amongst others, effector T cells (Teffs), which primarily consist of CD4+ helper T cells (Th1, Th2 and Th17 cells), CD8+ cytotoxic T cells and CD4+ regulatory T cells (Tregs) (for review, see [44]). Whereas Teffs perform proinflammatory immune functions, such as the killing of virus-infected and cancer cells by cytotoxic T cells or the activation of cytotoxic T cells and memory B cells by helper T cells, Tregs function to suppress the immune response [45]. In addition, Tregs play a pivotal role in enforcing the elimination of self-reactive T cells [46], and also B cells [47], that have escaped central tolerance. This occurs in the periphery and is considered an important part of the peripheral tolerance checkpoint. As a result, Tregs are essential for efficient enforcement of self-tolerance, thereby avoiding autoimmunity, as well as to prevent immune responses against foreign antigens from becoming harmful [48].

T cell metabolism is tightly regulated to support the differentiation and specialised functions of these distinct T cell subpopulations [49]. Naïve T cells, which primarily depend on OxPhos for energy production, upon differentiating into effector T cells, for example, typically show a metabolic shift to increased dependency upon glucose uptake and aerobic glycolysis, during which glucose is converted to lactate in the cytosol [49,50]. Although energetically less efficient than OxPhos, aerobic glycolysis is crucial for the maintenance of effector function such as rapid proliferation and especially the release of proinflammatory cytokines [50,51,52]. Indeed, genetic reduction of mitochondrial respiration in mice has been shown to further enhance aerobic glycolysis in activated Teffs and biased their differentiation towards proinflammatory subtypes [53]. The accumulation of lactate, the end product resulting from increases in both aerobic and anaerobic glycolysis, is considered a marker of T cell activation since high levels accumulate in areas of chronic inflammation, which in turn may influence the direction of the inflammatory process [54,55].

Stimulated Tregs, on the other hand, have been shown to require fatty acid oxidation in addition to glycolysis for their proliferation and migration [56,57,58,59]. However, the stability of the lineage-specific transcription factor Forkhead box P3 (FoxP3), which is vital for the suppressive capacity of Tregs, is critically dependent on OxPhos [56,60,61]. FoxP3 expression suppresses glycolysis and upregulates OxPhos, whereas an increase in glycolytic activity or ablation of mitochondrial respiration has been shown to reduce the Treg suppressive capacity [60,62,63].

This is similarly seen in other immune cells including B cells, where upon stimulation rather than increasing glycolysis, both OxPhos and TCA cycle activity, as well as nucleotide biosynthesis, are increased [64]. Likewise, macrophages can adopt either proinflammatory activated phenotypes that are dependent upon glycolysis [65,66], or anti-inflammatory phenotypes where OxPhos is the main metabolic source of energy along with fatty acid oxidation [67].

### 3.2. The Role of Lymphocytes in MS and EAE

Although the processes involved in the development of autoimmune diseases are not fully understood, they clearly involve an impairment in lymphocytic activity, with an imbalance in the effector and regulatory arms of the adaptive immune response. According to the ‘outside-in’ hypothesis of MS, acute CNS lesions in early relapsing–remitting disease are populated by peripherally activated autoreactive lymphocytes, including T cells of the CD4+ and CD8+ lineages, as well as B cells that invade the brain via a compromised blood–brain barrier [10]. These cells contribute to inflammation and tissue degeneration through the release of proinflammatory cytokines, the production of auto-antibodies and the recruitment and activation of additional immune cells, including macrophages and microglia [10]. The importance of T and B lymphocytes in MS pathophysiology is highlighted by the success of immunosuppressive and immunomodulatory therapies that target these cells in the treatment of RRMS [1]. Although these treatments cannot successfully slow disease progression in SPMS or PPMS, peripheral immune cells may still contribute to inflammation in progressive disease stages, albeit behind a closed blood–brain barrier [1].

That effector T and B cells are inefficiently suppressed by Tregs during autoimmune diseases is primarily supported by the role of Tregs in experimental models of autoimmunity. These include their ability to suppress autoimmune responses in murine models of autoimmune diabetes [68] and collagen-induced arthritis [69], as well as the animal model of MS, experimental autoimmune encephalomyelitis (EAE) [70,71]. Although it is currently unclear whether Treg numbers are altered in MS, with mixed reports available, there appears to be a clearer consensus that the suppressive capacity of MS patient-derived Tregs is impaired [72,73,74,75].

Due to the metabolic alterations involved in the differentiation of lymphocytes according to their functions and the importance of balancing both effector and regulatory lymphocytic immune pathways, it has been suggested that an impairment in lymphocytic metabolism may be responsible for this imbalance. Indeed, altered mitochondrial metabolism in lymphocytes has been observed in several autoimmune and neurodegenerative disorders, including rheumatoid arthritis, systemic lupus erythematosus, Alzheimer’s and Parkinson’s diseases [76,77,78,79] and may also contribute to MS and EAE as outlined below.

## 4. Metabolic Alterations in Lymphocytes of MS Patients and in EAE Mice

While mitochondrial dysfunction in the CNS of MS patients is now well established (see Section 2), recent research has also found metabolic and mitochondrial alterations in peripheral blood mononuclear cells (PBMCs) of MS patients, especially in CD4+ T cells and in T cells derived from the spleen and CNS of EAE mice. Given the prominent role of lymphocytes in MS pathophysiology and the influence of metabolism on immune cell function, gaining a detailed understanding of MS/EAE immunometabolism is of great interest, not least because it may provide clues for specific novel targets for treatment. Recent evidence for metabolic alterations in PBMCs of MS patients (see Table 1) and in T cells derived from EAE mice is summarised below.

### 4.1. Mitochondrial OxPhos Activity Is Decreased in PBMCs of MS Patients and in EAE

A number of studies provide evidence for changes in mitochondrial OxPhos activity in peripheral lymphocytes of RRMS patients. For example, a significant decrease in the mitochondrial respiratory control ratio in CD4+ cells of treatment-naïve RRMS patients compared to healthy controls has been reported indicating a reduced coupling efficiency between electron transport through the ETC and ATP production [80,81]. Mitochondrial OCR has also been reported to be significantly lower in CD4+ T cells but not in CD8+ T cells of RRMS patients in remission who received immunomodulatory therapy and in PBMCs of treatment-naïve RRMS patients [80,81,85,86,87]. This OCR reduction was accompanied by a significant decrease in the enzymatic activity of ETC complexes I and IV in CD4+ T cells and in whole PBMCs of untreated RRMS patients compared to healthy controls [80,81,91]. Furthermore, a significant reduction in the expression levels of respiratory chain complexes I, III and V in PBMCs of RRMS patients receiving various treatments and a decreased mitochondrial membrane potential that was particularly pronounced in patients with severe disease has also been observed [83,90]. In addition, RNA sequencing revealed significant changes in the transcriptome of CD4+ Tregs isolated from RRMS patients and specifically an enrichment in pathways associated with mitochondrial depolarisation and mitochondrial outer membrane permeabilization, indicative of dysfunctional mitochondrial metabolism in this specific T cell subpopulation [73].

To date, only one study has assessed mitochondrial OxPhos activity in progressive MS patients. Here, a decrease in the OCR of naïve CD4+ T cells, and to a lesser extent in memory CD4+ T cells, was seen in PPMS patients compared to healthy controls. This was accompanied by a lower mitochondrial mass and altered mitochondrial morphology. However, there was no reduction in OxPhos activity in CD4+ T cells of SPMS patients [89].

Analysis of these types of data needs to be performed carefully, however, since as outlined in Section 3.1, changes in T cell metabolism are part of the natural activation and differentiation of T cells. Therefore, any observed differences between healthy and MS patients may reflect the higher activation status of the T cells, as would be true during a period of infection even in a healthy control without an autoimmune condition. Therefore, animal models have been used to further elucidate whether altered lymphocytic metabolism really reflects potential pathogenic dysregulations and to explore whether modulation of these pathways could impact disease progression.

Accordingly, in one such EAE study, an increase in the mitochondrial OCR, accompanied by a decrease in the mitochondrial membrane potential, cytochrome c expression and reduced ATP levels were reported in Tregs isolated from draining lymph nodes of mice [73]. Interestingly, this was not only seen during active disease stages, but also prior to disease onset, suggesting that these changes in mitochondrial OxPhos activity may be involved in the subsequent development of disease. Additionally, an accumulation of mitochondria with aberrant morphology and cristae organisation were seen suggesting these observations are indicative of a mitochondrial pathology [73]. Since these alterations were only observed in FoxP3+ CD4+ T cells but not in FoxP3- CD4+ T cells, it suggests that mitochondrial dysfunctions might be restricted to the Treg subpopulation in EAE [73].

Overall, these findings suggest that mitochondrial OxPhos activity is decreased in PBMCs, especially in CD4+ T cells of RRMS patients, possibly due to a decrease in the expression or activity of several mitochondrial respiratory chain complexes. A similar decrease in OxPhos activity may also be present in PPMS patients, although this requires further investigation. Dysfunctional OxPhos has also been reported in EAE, although these alterations were restricted to the Treg compartment [73]. Since OxPhos has been shown to be crucial for the maintenance of Treg suppressive capacity, a more detailed analysis of this T cell subpopulation in human MS is warranted.

### 4.2. Oxidative Stress Is Increased in PBMCs of MS Patients and in EAE

In addition to the above evidence that favouring T cell OxPhos over glycolysis may promote the differentiation of T cells towards a less inflammatory phenotype, mitochondrial metabolism is also important for protecting the cell against ROS-mediated damage. ROS, such as superoxide, are by-products of mitochondrial metabolism and are scavenged by antioxidant defences under normal physiological conditions [23]. However, increased ROS production due to impairments in mitochondrial OxPhos activity or alterations in antioxidant capacity can result in oxidative damage that may further exacerbate changes in mitochondrial function in MS [80].

Indeed, CD4+ T cells of untreated RRMS patients showed oxidative protein damage that was accompanied by a significant reduction in the activity of two mitochondrial antioxidants, superoxide dismutase and glutathione peroxidase [80,81]. Reduced antioxidant capacity was also observed in the plasma of RRMS patients on immunomodulatory therapy together with a significant increase in superoxide production [83]. Furthermore, RNA sequencing revealed an enrichment in pathways associated with mitochondrial oxidative stress and DNA damage response in Tregs isolated from RRMS patients, which correlated with a decreased overall number of Tregs isolated from these patients [73].

In EAE, an increase in mtROS and a decrease in the mitochondrial antioxidant enzymes manganese superoxide dismutase and catalase in Tregs isolated from animals during presymptomatic disease stages compared to naïve Tregs or predisease nonTreg T cells has been found [73]. This was accompanied by an increase in DNA double-strand breaks and programmed cell death rates as indicated by an increased expression of caspase 3. Genetic ablation of Treg mitophagy in EAE mice revealed that increased mitochondrial oxidative stress and Treg cell death might be due to diminished clearance of mitochondria due to impaired lysosomal function [73]. On the other hand, scavenging of mitochondrial ROS using MitoTEMPO and genetic overexpression of the antioxidant enzyme catalase in Treg mitochondria significantly reduced mtROS production, Treg DNA damage and cell death, and restored lysosomal function. In addition, scavenging of mtROS reduced EAE disease severity, immune cell infiltration into the spinal cord and numbers of proinflammatory Th1 and Th17 cells [73].

Overall, it is conceivable that reduced antioxidant defences in peripheral lymphocytes of MS patients and in EAE-derived Tregs render mitochondria susceptible to oxidative damage, which may impair mitochondrial respiratory chain function subsequently contributing to the observed immunometabolic alterations. In addition, mitochondrial dysfunction in EAE Tregs and subsequent Treg cell death may further shift the T cell compartment towards proinflammatory subtypes.

### 4.3. Glycolytic Activity Is Altered in Lymphocytes of MS Patients and in EAE

The reduction in mitochondrial OxPhos activity in PBMCs of RRMS patients outlined above was accompanied by changes in glycolytic activity. It was shown, for example, that the activity of two key glycolytic enzymes, hexokinase and phosphofructokinase, and the expression of the main glucose importer GLUT1, were significantly increased in CD4+ T cells of treatment-naïve RRMS patients compared to healthy controls [80,81]. This was accompanied by increased lactate production in these cells and increased plasma lactate levels were also observed in RRMS patients on immunomodulatory treatments [80,83]. Similarly, using a metabolomics approach, a significant increase in serum glycolytic metabolites in RRMS patients, as well as increased ECAR in isolated PBMCs, was seen compared to healthy controls [88]. Another study found that ECAR was increased in CD4+ T cells of treatment-naïve RRMS patients during relapse but not during remission [84]. However, the same CD4+ T cells from relapsing RRMS patients also exhibited increased oxygen consumption, suggesting a general upregulation of T cell metabolism rather than a compensatory switch from OxPhos to aerobic glycolysis [84]. It is suggested that together, these results could be indicative of a glycolytic upregulation in CD4+ T cells of RRMS patients, possibly as a compensatory mechanism in response to impaired mitochondrial OxPhos or due to alterations in the metabolic programming and thus, the activation state of these cells [80].

In contrast, other studies reported a significantly decreased ECAR in CD4+ T cells isolated from untreated RRMS patients and from RRMS patients on immunomodulatory therapy during remission [82,85,87]. A decreased expression of GLUT1 and several glycolytic enzymes, including hexokinase, in CD4+ T cells of RRMS patients were also observed in the same sample [85]. It has additionally been shown that the defective glycolysis observed in CD4+ Tcons was associated with reduced induction of a specific Treg subpopulation and reduced Treg suppressive capacity and FoxP3 expression in untreated RRMS patients [82].

Similarly, ECAR has been found to be reduced in naïve and memory CD4+ T cells of PPMS but not SPMS patients, although surprisingly, GLUT1 expression and plasma lactate levels were significantly higher in PPMS samples [89]. Taken together, these results are suggestive of a general hypometabolic state, rather than a metabolic switch, at least in a subset of CD4+ T cells and possibly during distinct forms and phases of the disease.

While human studies have shown evidence of both an up- or a down-regulation in the glycolytic activity of MS-derived lymphocytes, there appears to be a clear consensus that glycolysis is upregulated in T cells isolated from EAE mice, as well as evidence that this might be involved in driving inflammation. This most likely reflects that most animal studies have treated Tregs and Teffs as separate compartments for analysis, unlike in the MS analyses.

For example, one study reported an upregulation of genes involved in glycolysis, including HIF-1α, PKM2 and GLUT1, in splenocytes isolated from EAE mice during active disease, compared to naïve control animals [92]. These metabolic changes were accompanied by an increase in proinflammatory Th17 cells and a downregulation of Tregs, suggesting that altered immunometabolism affects immune cell composition and thus inflammation in EAE. Similarly, it was shown that pathogenic Th17 cells had a higher ECAR, indicating an increase in aerobic glycolysis and also increased TCA cycle activity compared to nonpathogenic T cells [93]. A further study reported that the increased glycolytic activity associated with Th17 differentiation involved the activation of the transcription factor HIF1α by mTORC1 activity [94].

Glycolysis has also been shown to be upregulated in T cells isolated from the CNS of EAE mice. For example, it has been reported that proinflammatory Th17 cells isolated from the spinal cords of MOG-immunised mice showed above all an enrichment in pathways associated with glycolysis compared to homeostatic Th17 cells from the intestine [95]. Similarly, another study reported an upregulation of glycolytic activity in CD4+ and CD8+ T cells isolated from the spinal cord of EAE mice during peak disease [96]. This upregulation was reported in comparison to splenic T cells during EAE but did not evaluate whether splenic glycosylation was also altered in EAE by comparison to any non-EAE controls. However, the authors set out to evaluate the influence of the autoinflammatory environment of the CNS on the metabolic profile of T cells, rather than address the metabolism of active versus inactive T cells. They thus demonstrated that glycolytic changes are further enhanced upon T cells migrating to the CNS.

### 4.4. Modulation of Glycolytic Activity Ameliorates EAE

Many of the studies mentioned above reporting an increase in lymphocytic glycolysis in EAE also demonstrated the therapeutic potential of inhibiting glycolysis through both genetic ablation of key components of the glycolytic pathway or through pharmacological inhibition of key enzymes. Some of the experiments discussed in this review are summarised in Table 2.

For example, inhibition of glucose metabolism by inhibiting the first enzyme in the glycolytic pathway, hexokinase, was achieved through application of 2-deoxy-D-glucose (2-DG) and was shown to reduce EAE severity, as well as to upregulate Treg differentiation [94]. Another study employing 2-DG also reported it to protect mice from EAE through reducing CNS inflammation, as well as reporting a reduction in T cell infiltration and IFNγ release without affecting IL-17 levels [97]. The latter study also demonstrated similar effects through genetic ablation of Raptor, an essential component of mTORC1 signalling, which regulates the glycolytic rate. An alternative method of inhibiting hexokinase to suppress glycolysis was demonstrated by application of 3-bromopyruvic acid (3-BrPa), which also targets GAPDH resulting in the blockade of IFNγ release from Th1 cells, and to a lesser extent IL-17A produced by Th17 cells, and consequently dampened EAE severity [96].

Interestingly, genetic ablation of the glycolytic pathway enzyme, glucose phosphate isomerase (Gpi1), significantly reduced the number of inflammatory Th17 cells in EAE but had no significant effect on the number of homeostatic Th17 cells and Gpi1 deficient cells were unable to induce EAE indicating that Gpi1 is selectively required for proinflammatory Th17 cells [95]. The authors show that homeostatic Th17 cells are able to compensate for Gpi1 deficiency through increased activity of the pentose phosphate pathway and increased mitochondrial respiration, while proinflammatory Th17 cells cannot, possibly due to a more hypoxic environment during inflammation. A further study demonstrated that the common diabetic drug, metformin, could also reduce EAE severity through the reduction of both CNS immune cell infiltration and the ratio between proinflammatory Th17 and anti-inflammatory Tregs [92]. Although metformin is reported to function via multiple molecular mechanisms, since it lowers glucose production by the liver, it may be exerting its immunomodulatory effects through suppressing downstream glycolysis and subsequently favouring T cell differentiation towards a less proinflammatory phenotype.

Collectively, these data identify glycolysis as an important metabolic switch that drives T cell differentiation from quiescence towards a proinflammatory phenotype, thus aggravating autoimmunity. Since Teffs are reported to be more dependent upon glycolysis than Tregs [49,56], the strategy of targeting glycolysis may preferentially modulate the proinflammatory immune response. This hypothesis was explored by Gerriets et al. who identified pyruvate dehydrogenase (PDH) function to be important in balancing T cell dependence upon either glycolytic or oxidative metabolism, whereby PDH kinase 1 activity was associated with proinflammatory Th17 activity and PDH kinase 1 suppression increased Treg numbers [63]. In keeping with this observation, they further demonstrated that pharmacological inhibition of PDH kinase 1 in EAE decreased Th17 cells whilst increasing Tregs, thus ameliorating EAE severity.

### 4.5. Common RRMS Treatments Can Restore Metabolic Alterations in PBMCs of MS Patients

Several studies have found that common pharmacological treatments for RRMS can restore the observed immunometabolic alterations. Thus, 12 months of treatment with glatiramer acetate, a common first line treatment for RRMS, restored the respiratory control ratio, complex I and IV activity and mitochondrial membrane potential in CD4+ T-cells of previously untreated RRMS patients to levels comparable to those observed in healthy controls [81]. Glatiramer acetate treatment also normalised glycolytic enzyme activity, GLUT1 expression and lactate production, suggesting a treatment-induced reduction in glycolytic activity and recovery of mitochondrial OxPhos capacity in CD4+ T cells of RRMS patients [81].

Additionally, PBMCs isolated from RRMS patients receiving IFN-β did not show reduced levels of aerobic glycolysis, mitochondrial oxygen consumption and complex IV activity in contrast to cells of untreated patients, indicating that IFN-β treatment restores normal immunometabolic activity [85,91].

Finally, teriflunomide, an enzyme that blocks pyrimidine synthesis, was shown to restore glycolytic activity and mitochondrial OxPhos in CD4+ T cells of RRMS patients currently suffering a relapse to levels similar to those observed in healthy controls [84].

In contrast to restoring immunometabolic changes, another study found that in RRMS patients, dimethyl fumarate treatment supressed the antioxidant capacity of CD4+ and CD8+ T cells through modulation of mitochondrial metabolism resulting in an increase in mitochondrial ROS, in turn inducing a mitochondrial stress response and ultimately increased apoptosis, which especially affected memory T cells [86].

Taken together, these results show that several pharmacological treatments for RRMS are able to restore metabolic changes associated with MS. The mechanisms by which these treatments restore immunometabolism and whether this represents part of their therapeutic benefit or is merely a secondary effect (i.e., reflecting the lower lymphocyte activation status) should be investigated further. It is similarly unclear whether an impairment in T cell metabolism precedes the development of MS and thus represents an additional risk factor or whether this is a result of the heightened T cell activation occurring under autoinflammatory conditions.

## 5. Conclusions

Overall, the research summarised in this review provides evidence that metabolic alterations are a feature of MS and EAE, not only in the CNS, but also in peripheral lymphocytes. Primarily, changes in the balance between glycolysis and other metabolic processes, such as OxPhos, reflect the activation, proliferation and differentiation processes undertaken by T cells and other lymphocytes in order to allow the immune response to adapt to its required functions. As a result, it is harder in autoimmune diseases to determine whether observed metabolic changes reflect an intrinsic, aberrant response perhaps underlying an individual’s increased risk to developing MS or instead occurs as a result of the autoimmune environment already present. However, the fact that immunomodulatory therapies for MS appear to restore metabolic imbalances suggests that these changes may mainly reflect the overactive status present under autoimmune conditions.

That said, there is evidence of mitochondrial stress and damage in MS lymphocytes, such as reduced antioxidant capacity and increased oxidative damage, as well as upregulation of genetic programmes involved in mitochondrial oxidative stress, DNA damage and cell death, which are indicative of mitochondrial dysfunction rather than an (adaptive) upregulation of immune cell metabolism under inflammatory conditions. However, further work is needed to determine the influence of lymphocytic oxidative stress on subsequent metabolic switches within autoimmune lymphocytes.

In this light, data showing that in animal models of MS the metabolic balance of lymphocytes can be modified pharmacologically to augment the disease progression is interesting. Although this does not answer the question whether the metabolic ‘imbalance’ characteristic of a proinflammatory response is a cause or effect of MS development, its role in the progression of autoimmunity is clear. Increasing the dependency of lymphocytes on OxPhos rather than aerobic glycolysis has been shown by several groups to favour the function of Tregs over Teffs and may prove to be an interesting concept for promoting remission over relapses in MS patients.

It should be noted, however, that comparing animal models with MS must be done with care. Firstly, the studies performed have taken different approaches where MS patient studies on the whole have not differentiated between Teffs and Tregs, instead usually analysing pooled PBMCs or total CD4+ T cells, unlike in EAE, where subpopulation distinctions have been taken into account. Secondly, T cell subpopulations can differ significantly between mice and humans [98,99] and thus direct comparisons must be accordingly adjusted. Thirdly, although metabolic reprogramming in human and murine Tregs shows many similarities, there are also important differences that need to be considered since human and mice Tregs may show differences in their dependency upon glycolysis [82]. Thus, impaired glycolysis in MS patients has been shown to result in reduced FoxP3 expression in Tregs impeding their suppressive function [60,62,63], while inhibiting glycolysis in EAE mice reduced disease burden and the release of proinflammatory cytokines [82]. Nonetheless, the discussed alterations in T cell metabolism in human MS patients may impact on T cell subpopulation composition, specifically on the number of proinflammatory subtypes, and it should be further researched whether this may be a driver of disease progression or rather a secondary consequence of other pathogenic mechanisms. This is especially relevant with view to research showing potentially reduced Treg numbers and reduced Treg suppressive capacity in MS [72,73,100].

Furthermore, variations between MS patient studies render interpretations of results difficult. In contrast to reports of a switch from OxPhos to glycolysis [80,81], some studies found a decrease in both mitochondrial OxPhos and glycolytic activity, suggesting a general hypometabolic state of peripheral lymphocytes in MS [85]. These contradictory findings may partially be explained by the vast heterogeneity of patient samples, as well as the differing ratios of Teffs to Tregs that may exist at different disease stages. Thus, although some studies specifically assessed lymphocytes from RRMS patients in remission [84,87], most did not take disease activity into account. However, as shown by Klotz and colleagues, differences in immunometabolism in relapsing MS patients compared to patients in remission exist [84] and a potential correlation between disease activity and the lymphocyte’s metabolic profile should be further explored. A further complication in interpreting these results also stems from the fact that several studies assessed samples from patients on various immunomodulatory treatments. Some of these treatments have been shown to restore the observed metabolic alterations in peripheral lymphocytes of MS patients (see Section 4.5) and future studies should take this effect into account, especially when the sample size is small.

Finally, so far only one study has specifically assessed metabolic activity in PBMCs in progressive MS and found that immunometabolism differed between SPMS and PPMS patients [89]. Future research should further assess metabolic alterations in progressive disease and compare these to immunometabolic profiles during the relapsing stage of MS. Pathophysiological differences between RRMS and progressive disease are highlighted by the varying degree of CNS infiltration by activated lymphocytes and the different effects of immunomodulatory treatments and may also be reflected by distinct metabolic alterations [1]. In addition, gaining a detailed understanding of immunometabolism in PPMS and SPMS may help to further elucidate whether they represent diseases with distinct pathogeneses and possibly provide clues for the development of treatments specifically for progressive forms of MS [1]. Hints that this might be the case are derived from studies demonstrating a strong Th1/IFNγ-driven immune response in progressive disease [11,12], which may reflect the metabolic status of the immune cells involved.

Taken together, current research supports the emerging concept of altered mitochondrial function and cellular energy metabolism in peripheral immune cells as a characteristic feature in MS and its animal model. Further research is required to assess whether these immunometabolic alterations differ between distinct forms of the human disease, whether they are specific to CD4+ T cells, whether they influence lymphocytic activation and function and whether they correlate with disease activity in RRMS (see Box 1).

Box 1 **Open questions regarding MS lymphocytic metabolism.**

1. Do immunometabolic alterations in CD4+ T-cells contribute to disease progression or are they secondary to other MS pathophysiological processes?
2. Are immunometabolic alterations present in all CD4+ T cells or are there differences in Teffs and Tregs as suggested by EAE studies? Can similar changes be observed in other immune cells implicated in MS pathophysiology, such as B-cells?
3. What is the effect of altered metabolism on lymphocytic activation state and function, specifically with regard to Treg suppressive capacity?
4. Is there a correlation between immunometabolic alterations in PBMCs and RRMS disease activity and does it differ between RRMS and progressive forms of the disease?
5. What are the underlying molecular mechanisms responsible for altered immunometabolism in PBMCs? What is the role of ROS?
6. What is the mechanism by which common pharmacological treatments restore altered immunometabolism in peripheral lymphocytes?

## Figures and Tables

**Table 1:** Research providing evidence for altered immunometabolism in MS patients.

Reference	Sample Size (MS Phenotype Where Known)	Medication	Cell Type	Main Findings (MS Patients Compared to Controls)
Immunometabolism in RRMS				
Alissafi et al., 2020 [73]	11 MS (RRMS, all active)	N/A	Tregs	- Decreased Treg numbers. - RNAseq revealed increased expression of genes involved in pathways associated with mitochondrial dysfunction, oxidative stress and DNA damage.
De Riccardis et al., 2015 [80]	25 MS (RRMS) 8 HC	25 without treatment	CD4+ T cells	- Decreased OCR and complex I and IV activity. - Increased hexokinase and phosphofructokinase activity and GLUT1 expression. - Increased oxidative damage and reduced antioxidant capacity (GPX, SOD).
De Riccardis et al., 2016 [81]	20 MS (RRMS) 20 HC	20 without treatment, 20 GA *	CD4+ T cells	- Decreased OCR and complex I and IV activity. - Reduced antioxidant capacity (CAT, GPX and SOD). - Increased hexokinase and phosphofructokinase activity, GLUT1 expression and extracellular lactate release. - Alterations could be reversed after 12 months of GA treatment.
De Rosa et al., 2015 [82]	MS (RRMS) HC (unclear participant numbers)	Without treatment	CD4+ conventional T cells	- RRMS Tcons showed impaired glycolytic activity. - Induced Tregs derived from Tcons of RRMS patients had reduced suppressive capacity and FoxP3 expression.
Gonzalo et al., 2019 [83]	34 MS (RRMS) 24 HC	26 various IM, 8 without treatment	PBMCs	- Reduced complex I, III and V expression. - Increased plasma lactate concentration. - Increased ROS production and decreased plasma antioxidant capacity.
Klotz et al., 2019 [84]	49 MS (RRMS; 25 in remission, 24 with relapse) 24 HC	49 without treatment, 49 TF **	CD4+ T cells	- Enhanced OCR and ECAR in MS patients during relapse, but not in those in remission. - TF reduced OCR and ECAR in MS patients.
La Rocca et al., 2017 [85]	71 MS (RRMS) 57 HC	32 without treatment, 39 IFN-β	PBMCs CD4+ T cells	- Reduced OCR and ECAR in treatment-naïve MS patients. - Reduced expression of glycolytic enzymes and GLUT1 in treatment-naïve MS patients. - No differences in IFN-β-treated MS patients.
Liebmann et al., 2021 [86]	42 MS (RRMS) 15 HC	DMF	CD4+ T cells CD8+ T cells	- DMF reduces T cell antioxidant capacities, increases mtROS, MMP and apoptosis in CD4+ and CD8+ T cells. - These effects are more pronounced in memory T cells.
Tänzer, 2019 [87]	31 MS (RRMS, in remission) 31 HC	27 various IM, 4 without treatment	CD4+ T cells CD8+ T cells	- Reduced OCR and ECAR in CD4+ T-cells. - No differences in CD8+ T-cells.
Zahoor et al., 2022 [88]	35 MS (RRMS) 14 HC	N/A	PBMCs	- Serum metabolomics show increased glycolysis. - Increased basal ECAR.
Immunometabolism in Progressive MS				
De Biasi et al., 2019 [89]	53 MS (22 PPMS, 31 SPMS) 20 HC	53 without treatment	CD4+ T cells (naïve and memory cells)	- Decreased OCR and ECAR in naïve and memory CD4+ T-cells of PPMS patients. - No difference in OCR and ECAR in SPMS patients.
Immunometabolism in unspecified MS subtypes				
Armon-Omer et al., 2020 [90]	62 MS 83 HC	49 various IM, 13 without treatment	PBMCs	- Decreased MMP, which was stronger in patients with moderate-severe disease compared to those with mild disease.
Hargreaves et al., 2018 [91]	11 MS 24 HC	4 IFN-β, 7 without treatment	PBMCs	- Reduced complex IV activity in treatment-naïve MS patients. - No differences in IFN-β-treated MS patients.

CAT, catalase; DMF, dimethyl fumarate; ECAR, extracellular acidification rate; GA, glatiramer acetate; GPX, glutathione peroxidase; HC, healthy controls; IFN-β, interferon-beta; IM, immunomodulatory treatment; iTreg, inducible Tregs; MMP, mitochondrial membrane potential; MS, multiple sclerosis; N/A, not applicable; OCR, oxygen consumption rate; PPMS, primary progressive MS; RRMS, relapsing–remitting MS; SOD, superoxide dismutase; SPMS, secondary progressive MS; TF, teriflunomide. * Within-subjects design; untreated patients subsequently received 12 months of GA treatment; ** within-subjects design; untreated patients subsequently received 6 months of TF treatment.

**Table 2:** Amelioration of EAE through metabolic modulation of lymphocytes.

Reference	Modulation	Effects
Alissafi et al., 2020 [73]	Genetic overexpression of mitochondria-specific superoxide scavenger specifically in Tregs.	Protected Treg mitochondria from oxidative stress and reduced Th1 and Th17 activity.
Gerriets et al., 2015 [63]	DCA inhibition of PDHK1.	Decreased Th17 and increased Tregs; protected against EAE.
Karmaus et al., 2019 [97]	2-DG to inhibit hexokinase; Raptor knockout.	Reduced CNS inflammation.
Seki et al., 2017 [96]	3-BrPa inhibition of hexokinase and GAPDH.	Blocked Th1 release of IFNγ and Th17 release of IL-17; reduced EAE severity.
Shi et al., 2011 [94]	2-DG to inhibit hexokinase.	Increased Treg differentiation, and reduced EAE severity.
Sun et al., 2016 [92]	Metformin to inhibit AMPK.	Attenuation of EAE, Th17 proliferation, CNS immune cells infiltration and the release of proinflammatory cytokines; increased Treg proliferation and the release of anti-inflammatory cytokines.
Wagner et al., 2021 [97]	Disruption of polyamine pathway.	Increased frequency of Tregs and alleviated EAE symptoms.
Wu et al., 2020 [95]	Gpi1 knockout to suppress glycolysis.	Demonstrated requirement of proinflammatory Th17 cells for EAE induction.
Zahoor et al., 2022 [88]	2-DG to inhibit hexokinase.	Decreased disease severity, immune cell infiltration into the CNS, demyelination and the release of proinflammatory cytokines.

2-DG, 2-deoxy-D-glucose; 3-BrPa, 3-bromopyruvic acid; AMPK, AMP-activated protein kinase; DCA, dichloroacetate; EAE, experimental autoimmune encephalomyelitis; GAPDH, glyceraldehyde-3-phosphate dehydrogenase; Gpi1, glucose phosphate isomerase; PDHK1, pyruvate dehydrogenase kinase 1.

## Data Availability

Not applicable.

## References

[1] Lassmann H. (2018). Pathogenic Mechanisms Associated with Different Clinical Courses of Multiple Sclerosis. Front. Immunol..

[2] Walton C., King R., Rechtman L., Kaye W., Leray E., Marrie R.A., Robertson N., La Rocca N., Uitdehaag B., van der Mei I. (2020). Rising prevalence of multiple sclerosis worldwide: Insights from the Atlas of MS, third edition. Mult. Scler..

[3] Wallin M.T., Culpepper W.J., Nichols E., Bhutta Z.A., Gebrehiwot T.T., Hay S.I., Khalil I.A., Krohn K.J., Liang X., Naghavi M. (2019). Global, regional, and national burden of multiple sclerosis 1990–2016: A systematic analysis for the Global Burden of Disease Study 2016. Lancet Neurol..

[4] Disanto G., Berlanga A.J., Handel A.E., Para A.E., Burrell A.M., Fries A., Handunnetthi L., De Luca G.C., Morahan J.M. (2010). Heterogeneity in multiple sclerosis: Scratching the surface of a complex disease. Autoimmune Dis..

[5] Kister I., Bacon T.E., Chamot E., Salter A.R., Cutter G.R., Kalina J.T., Herbert J. (2013). Natural history of multiple sclerosis symptoms. Int. J. MS Care.

[6] Belbasis L., Bellou V., Evangelou E., Ioannidis J.P., Tzoulaki I. (2015). Environmental risk factors and multiple sclerosis: An umbrella review of systematic reviews and meta-analyses. Lancet Neurol..

[7] International Multiple Sclerosis Genetics C., Beecham A.H., Patsopoulos N.A., Xifara D.K., Davis M.F., Kemppinen A., Cotsapas C., Shah T.S., Spencer C., Booth D. (2013). Analysis of immune-related loci identifies 48 new susceptibility variants for multiple sclerosis. Nat. Genet..

[8] International Multiple Sclerosis Genetics Consortium (2018). Electronic address, c. c. y. e.; International Multiple Sclerosis Genetics, C. Low-Frequency and Rare-Coding Variation Contributes to Multiple Sclerosis Risk. Cell.

[9] Dendrou C.A., Fugger L., Friese M.A. (2015). Immunopathology of multiple sclerosis. Nat. Rev. Immunol..

[10] Baecher-Allan C., Kaskow B.J., Weiner H.L. (2018). Multiple Sclerosis: Mechanisms and Immunotherapy. Neuron.

[11] Frisullo G., Plantone D., Marti A., Iorio R., Damato V., Nociti V., Patanella A.K., Bianco A., Batocchi A.P. (2012). Type 1 immune response in progressive multiple sclerosis. J. Neuroimmunol..

[12] Balashov K.E., Smith D.R., Khoury S.J., Hafler D.A., Weiner H.L. (1997). Increased interleukin 12 production in progressive multiple sclerosis: Induction by activated CD4+ T cells via CD40 ligand. Proc. Natl. Acad. Sci. USA.

[13] Karni A., Abraham M., Monsonego A., Cai G., Freeman G.J., Hafler D., Khoury S.J., Weiner H.L. (2006). Innate immunity in multiple sclerosis: Myeloid dendritic cells in secondary progressive multiple sclerosis are activated and drive a proinflammatory immune response. J. Immunol..

[14] Dargahi N., Katsara M., Tselios T., Androutsou M.E., de Courten M., Matsoukas J., Apostolopoulos V. (2017). Multiple Sclerosis: Immunopathology and Treatment Update. Brain Sci..

[15] Derfuss T., Mehling M., Papadopoulou A., Bar-Or A., Cohen J.A., Kappos L. (2020). Advances in oral immunomodulating therapies in relapsing multiple sclerosis. Lancet Neurol..

[16] Witte M.E., Mahad D.J., Lassmann H., van Horssen J. (2014). Mitochondrial dysfunction contributes to neurodegeneration in multiple sclerosis. Trends Mol. Med..

[17] Shulman R.G., Rothman D.L., Behar K.L., Hyder F. (2004). Energetic basis of brain activity: Implications for neuroimaging. Trends Neurosci..

[18] Lunt S.Y., Vander Heiden M.G. (2011). Aerobic glycolysis: Meeting the metabolic requirements of cell proliferation. Annu. Rev. Cell. Dev. Biol..

[19] Brooks G.A. (2020). Lactate as a fulcrum of metabolism. Redox. Biol..

[20] Yin M., O’Neill L.A.J. (2021). The role of the electron transport chain in immunity. FASEB J..

[21] Brand M.D., Nicholls D.G. (2011). Assessing mitochondrial dysfunction in cells. Biochem. J..

[22] Connolly N.M.C., Theurey P., Adam-Vizi V., Bazan N.G., Bernardi P., Bolanos J.P., Culmsee C., Dawson V.L., Deshmukh M., Duchen M.R. (2018). Guidelines on experimental methods to assess mitochondrial dysfunction in cellular models of neurodegenerative diseases. Cell Death Differ..

[23] Zorov D.B., Filburn C.R., Klotz L.O., Zweier J.L., Sollott S.J. (2000). Reactive oxygen species (ROS)-induced ROS release: A new phenomenon accompanying induction of the mitochondrial permeability transition in cardiac myocytes. J. Exp. Med..

[24] Zhao R.Z., Jiang S., Zhang L., Yu Z.B. (2019). Mitochondrial electron transport chain, ROS generation and uncoupling (Review). Int. J. Mol. Med..

[25] TeSlaa T., Teitell M.A. (2014). Techniques to monitor glycolysis. Methods Enzymol..

[26] van Horssen J., van Schaik P., Witte M. (2019). Inflammation and mitochondrial dysfunction: A vicious circle in neurodegenerative disorders?. Neurosci. Lett..

[27] Campbell G., Mahad D.J. (2018). Mitochondrial dysfunction and axon degeneration in progressive multiple sclerosis. FEBS Lett..

[28] Barcelos I.P., Troxell R.M., Graves J.S. (2019). Mitochondrial Dysfunction and Multiple Sclerosis. Biology.

[29] Blagov A.V., Sukhorukov V.N., Orekhov A.N., Sazonova M.A., Melnichenko A.A. (2022). Significance of Mitochondrial Dysfunction in the Progression of Multiple Sclerosis. Int. J. Mol. Sci..

[30] Fischer M.T., Sharma R., Lim J.L., Haider L., Frischer J.M., Drexhage J., Mahad D., Bradl M., van Horssen J., Lassmann H. (2012). NADPH oxidase expression in active multiple sclerosis lesions in relation to oxidative tissue damage and mitochondrial injury. Brain.

[31] Lu F., Selak M., O’Connor J., Croul S., Lorenzana C., Butunoi C., Kalman B. (2000). Oxidative damage to mitochondrial DNA and activity of mitochondrial enzymes in chronic active lesions of multiple sclerosis. J. Neurol. Sci..

[32] Mahad D., Ziabreva I., Lassmann H., Turnbull D. (2008). Mitochondrial defects in acute multiple sclerosis lesions. Brain.

[33] Mahad D.J., Ziabreva I., Campbell G., Lax N., White K., Hanson P.S., Lassmann H., Turnbull D.M. (2009). Mitochondrial changes within axons in multiple sclerosis. Brain.

[34] Witte M.E., Bo L., Rodenburg R.J., Belien J.A., Musters R., Hazes T., Wintjes L.T., Smeitink J.A., Geurts J.J., De Vries H.E. (2009). Enhanced number and activity of mitochondria in multiple sclerosis lesions. J. Pathol..

[35] Zambonin J.L., Zhao C., Ohno N., Campbell G.R., Engeham S., Ziabreva I., Schwarz N., Lee S.E., Frischer J.M., Turnbull D.M. (2011). Increased mitochondrial content in remyelinated axons: Implications for multiple sclerosis. Brain.

[36] Craner M.J., Newcombe J., Black J.A., Hartle C., Cuzner M.L., Waxman S.G. (2004). Molecular changes in neurons in multiple sclerosis: Altered axonal expression of Nav1.2 and Nav1.6 sodium channels and Na+/Ca2+ exchanger. Proc. Natl. Acad. Sci. USA.

[37] Campbell G., Licht-Mayer S., Mahad D. (2019). Targeting mitochondria to protect axons in progressive MS. Neurosci. Lett..

[38] Broadwater L., Pandit A., Clements R., Azzam S., Vadnal J., Sulak M., Yong V.W., Freeman E.J., Gregory R.B., McDonough J. (2011). Analysis of the mitochondrial proteome in multiple sclerosis cortex. Biochim. Biophys. Acta.

[39] Campbell G.R., Ziabreva I., Reeve A.K., Krishnan K.J., Reynolds R., Howell O., Lassmann H., Turnbull D.M., Mahad D.J. (2011). Mitochondrial DNA deletions and neurodegeneration in multiple sclerosis. Ann. Neurol..

[40] Dutta R., McDonough J., Yin X., Peterson J., Chang A., Torres T., Gudz T., Macklin W.B., Lewis D.A., Fox R.J. (2006). Mitochondrial dysfunction as a cause of axonal degeneration in multiple sclerosis patients. Ann. Neurol..

[41] Witte M.E., Nijland P.G., Drexhage J.A., Gerritsen W., Geerts D., van Het Hof B., Reijerkerk A., de Vries H.E., van der Valk P., van Horssen J. (2013). Reduced expression of PGC-1alpha partly underlies mitochondrial changes and correlates with neuronal loss in multiple sclerosis cortex. Acta Neuropathol..

[42] Pandit A., Vadnal J., Houston S., Freeman E., McDonough J. (2009). Impaired regulation of electron transport chain subunit genes by nuclear respiratory factor 2 in multiple sclerosis. J. Neurol. Sci..

[43] Chapman N.M., Boothby M.R., Chi H. (2020). Metabolic coordination of T cell quiescence and activation. Nat. Rev. Immunol..

[44] Kumar B.V., Connors T.J., Farber D.L. (2018). Human T Cell Development, Localization, and Function throughout Life. Immunity.

[45] Benova K., Hanckova M., Koci K., Kudelova M., Betakova T. (2020). T cells and their function in the immune response to viruses. Acta Virol..

[46] Sakaguchi S., Yamaguchi T., Nomura T., Ono M. (2008). Regulatory T cells and immune tolerance. Cell.

[47] Kinnunen T., Chamberlain N., Morbach H., Cantaert T., Lynch M., Preston-Hurlburt P., Herold K.C., Hafler D.A., O’Connor K.C., Meffre E. (2013). Specific peripheral B cell tolerance defects in patients with multiple sclerosis. J. Clin. Investig..

[48] Goswami T.K., Singh M., Dhawan M., Mitra S., Emran T.B., Rabaan A.A., Mutair A.A., Alawi Z.A., Alhumaid S., Dhama K. (2022). Regulatory T cells (Tregs) and their therapeutic potential against autoimmune disorders—Advances and challenges. Hum. Vaccin. Immunother..

[49] Chapman N.M., Chi H. (2022). Metabolic adaptation of lymphocytes in immunity and disease. Immunity.

[50] Menk A.V., Scharping N.E., Moreci R.S., Zeng X., Guy C., Salvatore S., Bae H., Xie J., Young H.A., Wendell S.G. (2018). Early TCR Signaling Induces Rapid Aerobic Glycolysis Enabling Distinct Acute T Cell Effector Functions. Cell Rep..

[51] Chang C.H., Curtis J.D., Maggi L.B., Faubert B., Villarino A.V., O’Sullivan D., Huang S.C., van der Windt G.J., Blagih J., Qiu J. (2013). Posttranscriptional control of T cell effector function by aerobic glycolysis. Cell.

[52] Bantug G.R., Galluzzi L., Kroemer G., Hess C. (2018). The spectrum of T cell metabolism in health and disease. Nat. Rev. Immunol..

[53] Baixauli F., Acin-Perez R., Villarroya-Beltri C., Mazzeo C., Nunez-Andrade N., Gabande-Rodriguez E., Ledesma M.D., Blazquez A., Martin M.A., Falcon-Perez J.M. (2015). Mitochondrial Respiration Controls Lysosomal Function during Inflammatory T Cell Responses. Cell Metab..

[54] Manosalva C., Quiroga J., Hidalgo A.I., Alarcon P., Anseoleaga N., Hidalgo M.A., Burgos R.A. (2021). Role of Lactate in Inflammatory Processes: Friend or Foe. Front. Immunol..

[55] Pucino V., Certo M., Bulusu V., Cucchi D., Goldmann K., Pontarini E., Haas R., Smith J., Headland S.E., Blighe K. (2019). Lactate Buildup at the Site of Chronic Inflammation Promotes Disease by Inducing CD4(+) T Cell Metabolic Rewiring. Cell Metab..

[56] Yan Y., Huang L., Liu Y., Yi M., Chu Q., Jiao D., Wu K. (2022). Metabolic profiles of regulatory T cells and their adaptations to the tumor microenvironment: Implications for antitumor immunity. J. Hematol. Oncol..

[57] Kishore M., Cheung K.C.P., Fu H., Bonacina F., Wang G., Coe D., Ward E.J., Colamatteo A., Jangani M., Baragetti A. (2017). Regulatory T Cell Migration Is Dependent on Glucokinase-Mediated Glycolysis. Immunity.

[58] Cluxton D., Petrasca A., Moran B., Fletcher J.M. (2019). Differential Regulation of Human Treg and Th17 Cells by Fatty Acid Synthesis and Glycolysis. Front. Immunol..

[59] Michalek R.D., Gerriets V.A., Jacobs S.R., Macintyre A.N., MacIver N.J., Mason E.F., Sullivan S.A., Nichols A.G., Rathmell J.C. (2011). Cutting edge: Distinct glycolytic and lipid oxidative metabolic programs are essential for effector and regulatory CD4+ T cell subsets. J. Immunol..

[60] Fu Z., Ye J., Dean J.W., Bostick J.W., Weinberg S.E., Xiong L., Oliff K.N., Chen Z.E., Avram D., Chandel N.S. (2019). Requirement of Mitochondrial Transcription Factor A in Tissue-Resident Regulatory T Cell Maintenance and Function. Cell Rep..

[61] Weinberg S.E., Singer B.D., Steinert E.M., Martinez C.A., Mehta M.M., Martinez-Reyes I., Gao P., Helmin K.A., Abdala-Valencia H., Sena L.A. (2019). Mitochondrial complex III is essential for suppressive function of regulatory T cells. Nature.

[62] Angelin A., Gil-de-Gomez L., Dahiya S., Jiao J., Guo L., Levine M.H., Wang Z., Quinn W.J., Kopinski P.K., Wang L. (2017). Foxp3 Reprograms T Cell Metabolism to Function in Low-Glucose, High-Lactate Environments. Cell Metab..

[63] Gerriets V.A., Kishton R.J., Nichols A.G., Macintyre A.N., Inoue M., Ilkayeva O., Winter P.S., Liu X., Priyadharshini B., Slawinska M.E. (2015). Metabolic programming and PDHK1 control CD4+ T cell subsets and inflammation. J. Clin. Investig..

[64] Waters L.R., Ahsan F.M., Wolf D.M., Shirihai O., Teitell M.A. (2018). Initial B Cell Activation Induces Metabolic Reprogramming and Mitochondrial Remodeling. iScience.

[65] Meiser J., Kramer L., Sapcariu S.C., Battello N., Ghelfi J., D’Herouel A.F., Skupin A., Hiller K. (2016). Pro-inflammatory Macrophages Sustain Pyruvate Oxidation through Pyruvate Dehydrogenase for the Synthesis of Itaconate and to Enable Cytokine Expression. J. Biol. Chem..

[66] Freemerman A.J., Johnson A.R., Sacks G.N., Milner J.J., Kirk E.L., Troester M.A., Macintyre A.N., Goraksha-Hicks P., Rathmell J.C., Makowski L. (2014). Metabolic reprogramming of macrophages: Glucose transporter 1 (GLUT1)-mediated glucose metabolism drives a proinflammatory phenotype. J. Biol. Chem..

[67] Jha A.K., Huang S.C., Sergushichev A., Lampropoulou V., Ivanova Y., Loginicheva E., Chmielewski K., Stewart K.M., Ashall J., Everts B. (2015). Network integration of parallel metabolic and transcriptional data reveals metabolic modules that regulate macrophage polarization. Immunity.

[68] Boitard C., Yasunami R., Dardenne M., Bach J.F. (1989). T cell-mediated inhibition of the transfer of autoimmune diabetes in NOD mice. J. Exp. Med..

[69] Morgan M.E., Flierman R., van Duivenvoorde L.M., Witteveen H.J., van Ewijk W., van Laar J.M., de Vries R.R., Toes R.E. (2005). Effective treatment of collagen-induced arthritis by adoptive transfer of CD25+ regulatory T cells. Arthritis Rheum..

[70] Kohm A.P., Carpentier P.A., Anger H.A., Miller S.D. (2002). Cutting edge: CD4+CD25+ regulatory T cells suppress antigen-specific autoreactive immune responses and central nervous system inflammation during active experimental autoimmune encephalomyelitis. J. Immunol..

[71] Zhang X., Koldzic D.N., Izikson L., Reddy J., Nazareno R.F., Sakaguchi S., Kuchroo V.K., Weiner H.L. (2004). IL-10 is involved in the suppression of experimental autoimmune encephalomyelitis by CD25+CD4+ regulatory T cells. Int. Immunol..

[72] Verma N.D., Lam A.D., Chiu C., Tran G.T., Hall B.M., Hodgkinson S.J. (2021). Multiple sclerosis patients have reduced resting and increased activated CD4(+)CD25(+)FOXP3(+)T regulatory cells. Sci. Rep..

[73] Alissafi T., Kalafati L., Lazari M., Filia A., Kloukina I., Manifava M., Lim J.H., Alexaki V.I., Ktistakis N.T., Doskas T. (2020). Mitochondrial Oxidative Damage Underlies Regulatory T Cell Defects in Autoimmunity. Cell Metab..

[74] Putheti P., Pettersson A., Soderstrom M., Link H., Huang Y.M. (2004). Circulating CD4+CD25+ T regulatory cells are not altered in multiple sclerosis and unaffected by disease-modulating drugs. J. Clin. Immunol..

[75] Haas J., Hug A., Viehover A., Fritzsching B., Falk C.S., Filser A., Vetter T., Milkova L., Korporal M., Fritz B. (2005). Reduced suppressive effect of CD4+CD25high regulatory T cells on the T cell immune response against myelin oligodendrocyte glycoprotein in patients with multiple sclerosis. Eur. J. Immunol..

[76] Delbarba A., Abate G., Prandelli C., Marziano M., Buizza L., Arce Varas N., Novelli A., Cuetos F., Martinez C., Lanni C. (2016). Mitochondrial Alterations in Peripheral Mononuclear Blood Cells from Alzheimer’s Disease and Mild Cognitive Impairment Patients. Oxid Med. Cell Longev..

[77] Fearon U., Canavan M., Biniecka M., Veale D.J. (2016). Hypoxia, mitochondrial dysfunction and synovial invasiveness in rheumatoid arthritis. Nat. Rev. Rheumatol..

[78] Morel L. (2017). Immunometabolism in systemic lupus erythematosus. Nat. Rev. Rheumatol..

[79] Smith A.M., Depp C., Ryan B.J., Johnston G.I., Alegre-Abarrategui J., Evetts S., Rolinski M., Baig F., Ruffmann C., Simon A.K. (2018). Mitochondrial dysfunction and increased glycolysis in prodromal and early Parkinson’s blood cells. Mov. Disord..

[80] De Riccardis L., Rizzello A., Ferramosca A., Urso E., De Robertis F., Danieli A., Giudetti A.M., Trianni G., Zara V., Maffia M. (2015). Bioenergetics profile of CD4(+) T cells in relapsing remitting multiple sclerosis subjects. J. Biotechnol..

[81] De Riccardis L., Ferramosca A., Danieli A., Trianni G., Zara V., De Robertis F., Maffia M. (2016). Metabolic response to glatiramer acetate therapy in multiple sclerosis patients. BBA Clin..

[82] De Rosa V., Galgani M., Porcellini A., Colamatteo A., Santopaolo M., Zuchegna C., Romano A., De Simone S., Procaccini C., La Rocca C. (2015). Glycolysis controls the induction of human regulatory T cells by modulating the expression of FOXP3 exon 2 splicing variants. Nat. Immunol..

[83] Gonzalo H., Nogueras L., Gil-Sanchez A., Hervas J.V., Valcheva P., Gonzalez-Mingot C., Martin-Gari M., Canudes M., Peralta S., Solana M.J. (2019). Impairment of Mitochondrial Redox Status in Peripheral Lymphocytes of Multiple Sclerosis Patients. Front. Neurosci..

[84] Klotz L., Eschborn M., Lindner M., Liebmann M., Herold M., Janoschka C., Torres Garrido B., Schulte-Mecklenbeck A., Gross C.C., Breuer J. (2019). Teriflunomide treatment for multiple sclerosis modulates T cell mitochondrial respiration with affinity-dependent effects. Sci. Transl. Med..

[85] La Rocca C., Carbone F., De Rosa V., Colamatteo A., Galgani M., Perna F., Lanzillo R., Brescia Morra V., Orefice G., Cerillo I. (2017). Immunometabolic profiling of T cells from patients with relapsing-remitting multiple sclerosis reveals an impairment in glycolysis and mitochondrial respiration. Metabolism.

[86] Liebmann M., Korn L., Janoschka C., Albrecht S., Lauks S., Herrmann A.M., Schulte-Mecklenbeck A., Schwab N., Schneider-Hohendorf T., Eveslage M. (2021). Dimethyl fumarate treatment restrains the antioxidative capacity of T cells to control autoimmunity. Brain.

[87] Tänzer A. (2019). Molecular Mechanisms of Immunometabolic Dysfunction in Multiple Sclerosis.

[88] Zahoor I., Suhail H., Datta I., Ahmed M.E., Poisson L.M., Waters J., Rashid F., Bin R., Singh J., Cerghet M. (2022). Blood-based untargeted metabolomics in relapsing-remitting multiple sclerosis revealed the testable therapeutic target. Proc. Natl. Acad. Sci. USA.

[89] De Biasi S., Simone A.M., Bianchini E., Lo Tartaro D., Pecorini S., Nasi M., Patergnani S., Carnevale G., Gibellini L., Ferraro D. (2019). Mitochondrial functionality and metabolism in T cells from progressive multiple sclerosis patients. Eur. J. Immunol..

[90] Armon-Omer A., Neuman H., Sharabi-Nov A., Shahien R. (2020). Mitochondrial activity is impaired in lymphocytes of MS patients in correlation with disease severity. Mult. Scler. Relat. Disord..

[91] Hargreaves I., Mody N., Land J., Heales S. (2018). Blood Mononuclear Cell Mitochondrial Respiratory Chain Complex IV Activity Is Decreased in Multiple Sclerosis Patients: Effects of beta-Interferon Treatment. J. Clin. Med..

[92] Sun Y., Tian T., Gao J., Liu X., Hou H., Cao R., Li B., Quan M., Guo L. (2016). Metformin ameliorates the development of experimental autoimmune encephalomyelitis by regulating T helper 17 and regulatory T cells in mice. J. Neuroimmunol..

[93] Wagner A., Wang C., Fessler J., DeTomaso D., Avila-Pacheco J., Kaminski J., Zaghouani S., Christian E., Thakore P., Schellhaass B. (2021). Metabolic modeling of single Th17 cells reveals regulators of autoimmunity. Cell.

[94] Shi L.Z., Wang R., Huang G., Vogel P., Neale G., Green D.R., Chi H. (2011). HIF1alpha-dependent glycolytic pathway orchestrates a metabolic checkpoint for the differentiation of TH17 and Treg cells. J. Exp. Med..

[95] Wu L., Hollinshead K.E.R., Hao Y., Au C., Kroehling L., Ng C., Lin W.Y., Li D., Silva H.M., Shin J. (2020). Niche-Selective Inhibition of Pathogenic Th17 Cells by Targeting Metabolic Redundancy. Cell.

[96] Seki S.M., Stevenson M., Rosen A.M., Arandjelovic S., Gemta L., Bullock T.N.J., Gaultier A. (2017). Lineage-Specific Metabolic Properties and Vulnerabilities of T Cells in the Demyelinating Central Nervous System. J. Immunol..

[97] Karmaus P.W.F., Chen X., Lim S.A., Herrada A.A., Nguyen T.M., Xu B., Dhungana Y., Rankin S., Chen W., Rosencrance C. (2019). Metabolic heterogeneity underlies reciprocal fates of T(H)17 cell stemness and plasticity. Nature.

[98] Payne K.J., Crooks G.M. (2007). Immune-cell lineage commitment: Translation from mice to humans. Immunity.

[99] Seok J., Warren H.S., Cuenca A.G., Mindrinos M.N., Baker H.V., Xu W., Richards D.R., McDonald-Smith G.P., Gao H., Hennessy L. (2013). Genomic responses in mouse models poorly mimic human inflammatory diseases. Proc. Natl. Acad. Sci. USA.

[100] Schwarz A., Schumacher M., Pfaff D., Schumacher K., Jarius S., Balint B., Wiendl H., Haas J., Wildemann B. (2013). Fine-tuning of regulatory T cell function: The role of calcium signals and naive regulatory T cells for regulatory T cell deficiency in multiple sclerosis. J. Immunol..

